# Construction and Characterization of an *in-vivo* Linear Covalently Closed DNA Vector Production System

**DOI:** 10.1186/1475-2859-11-154

**Published:** 2012-12-06

**Authors:** Nafiseh Nafissi, Roderick Slavcev

**Affiliations:** 1 School of Pharmacy, Faculty of Science, University of Waterloo, Waterloo, Ontario, N2L 3G1, Canada

**Keywords:** Mini DNA vectors, Linear covalently closed plasmid vector, DNA vector integration, Non-viral gene delivery, “minicircles”, Bacteriophage PY54 Tel/*pal* recombination system, Bacteriophage N15 TelN/*telRL* recombination system, Bacteriophage P1 Cre/*loxP* recombination system, Bacterial engineering

## Abstract

**Background:**

While safer than their viral counterparts, conventional non-viral gene delivery DNA vectors offer a limited safety profile. They often result in the delivery of unwanted prokaryotic sequences, antibiotic resistance genes, and the bacterial origins of replication to the target, which may lead to the stimulation of unwanted immunological responses due to their chimeric DNA composition. Such vectors may also impart the potential for chromosomal integration, thus potentiating oncogenesis. We sought to engineer an *in vivo* system for the quick and simple production of safer DNA vector alternatives that were devoid of non-transgene bacterial sequences and would lethally disrupt the host chromosome in the event of an unwanted vector integration event.

**Results:**

We constructed a parent eukaryotic expression vector possessing a specialized manufactured multi-target site called “Super Sequence”, and engineered *E. coli* cells (R-cell) that conditionally produce phage-derived recombinase Tel (PY54), TelN (N15), or Cre (P1). Passage of the parent plasmid vector through R-cells under optimized conditions, resulted in rapid, efficient, and one step *in vivo* generation of mini lcc—linear covalently closed (Tel/TelN-cell), or mini ccc—circular covalently closed (Cre-cell), DNA constructs, separated from the backbone plasmid DNA. Site-specific integration of lcc plasmids into the host chromosome resulted in chromosomal disruption and 10^5^ fold lower viability than that seen with the ccc counterpart.

**Conclusion:**

We offer a high efficiency mini DNA vector production system that confers simple, rapid and scalable *in vivo* production of mini lcc DNA vectors that possess all the benefits of “minicircle” DNA vectors and virtually eliminate the potential for undesirable vector integration events.

## Background

The utility of any gene therapy strategy is defined by its balance between safety and effectiveness. While virus-derived vectors offer exceptional potential to target and deliver DNA cargo with high efficiency into the target cell, viral strategies often suffer in their safety profiling. Recent viral gene therapy-related patient mortalities in clinical trials highlight some of the safety issues attributed to the use of viral gene transfer systems that include, but are not limited to unwanted immune responses to viral capsid proteins, regeneration of virulent viruses, and insertional mutagenesis [1]. In contrast, non-viral strategies based on naked, lipoplexed or polyplexed plasmid DNA (pDNA) vectors generally offer safer gene therapy, vaccine design, and drug delivery approaches. Plasmid DNA vectors are relatively easy to generate and store and offer tremendous design capacity. Several major barriers need to be considered in order to develop non-viral gene delivery systems as a therapeutic product to be safely administered *in vivo*. A successful transgene delivery system depends on the entrance of the DNA vector into the mammalian host nucleus and expression of the encoded transgene(s). While simple in theory, several cellular barriers must be overcome in practice. While travelling in the extracellular surroundings, vectors must be bio- and immuno-compatible and avoid degradation by serum nucleases and immune detection by phagocytes. Plasma nucleases digest the unprotected DNA within just a few minutes, so DNA vectors need to rapidly cross the plasma membrane of target cells. This is further complicated by the fact that the plasma membrane is composed of dense lipoprotein barriers that intrinsically inhibit efficient DNA translocation. Strategies to overcome this barrier include complexing DNA vectors with synthetic nanoparticles to form a structure similar to the plasma membrane [2] or receptor-mediated endocytosis; *i.e.* targeted liposomes [3]. However, while non-viral gene delivery techniques work toward efficiency of DNA delivery, they generally prove poor in the delivery of pDNA vectors to the nuclear compartment. Many techniques are currently being investigated to enhance levels of non-viral gene transfer by targeting vectors to the nucleus. These techniques include modification of plasmids with DNA nuclear targeting sequences (DTS), covalent linkage of nuclear localization signals (NLS) to the plasmid DNA constructs, and attachment of import receptors such as karyopherins, to vectors that promote uptake through the nuclear membrane pore complex (NPC) [2,4,5]. Modification of DNA with NLS-conjugates seems to result in highly efficient expression of linear, but not circular DNA, in combination with liposomal delivery vectors [6]. This difference may be attributed to charge per unit ratio of linear versus supercoiled circular DNA and provides yet another intriguing opportunity for lcc vectors [7].

In addition to the aforementioned challenges, conventional non-viral gene delivery approaches may lead to unwanted immunological responses and oncogenesis, imparted by the presence of bacterial genetic elements in plasmid DNA constructs. These include prokaryotic origins of replication, antibiotic resistance genes, as well as high-frequency immunostimulatory CpG motifs that activate Toll-like receptors in mammalian hosts [8]. In order to improve the immuno-compatibily and durability of plasmid DNA vectors, a new generation of DNA vectors have been constructed that exploit the bacteriophage λ integrase (Int)-*attP* or P1-derived Cre-*loxP* site-specific recombination systems to generate mini ccc DNA vectors [9]. These “minicircles” provide safer minimized transgene vectors by removing unwanted prokaryotic elements, thus enhancing bio- and immuno-compatibility in the mammalian host [10]. The smaller size compared to the parental plasmid backbone also confers improved extracellular and intracellular bioavailability leading to efficient gene delivery and hence, improved gene expression [11].

A second group of modified vectors offering great promise are linear covalently closed (lcc) plasmid DNA vectors. Aside from the obvious topological differences, lcc double-stranded DNA molecules are torsion-free as they are not subject to gyrase-directed negative supercoiling, and as such possess the properties of linear DNA [12]. However, lcc DNA is not subject to ExoV exonuclease activity in prokaryotes due to covalent linkage of linear ends, preventing degradation of the lcc pDNA vector. Lcc DNA vectors have been constructed by various *in vitro* strategies including the capping of PCR products, and the “minimalistic immunogenic defined gene expression (MIDGE)” vectors. MIDGE is generated by the digestion of both prokaryotic and eukaryotic backbones after isolation of plasmid from bacterial cells, followed by ligation of the therapeutic expression cassette into hairpin sequences for end-refilling [13]. This technology has shown promising results in various applications including the development of a *Leishmania* DNA vaccine [14] and a colon carcinoma treatment [15]. MIDGE vectors have also demonstrated up to 17 fold improved transgene expression profile *in vivo* in some tissues, compared to conventional plasmid DNA vectors [16]. Thus, lcc DNA vectors may in fact outperform their circular counterparts with respect to expression efficiency and bioavailability. However, large-scale production of lcc DNA vectors via existing multistep *in vitro* processes requires considerable time and financial cost.

*E. coli* phage N15 was the first discovered phage to exist in its lysogenic (prophage) state as a linear covalently closed (lcc) plasmid [17] that is actively partitioned to daughter cells [18]. The lcc conformation is conferred by the cleaving-joining activity of the protelomerase protein (Prokaryotic Telomerase), TelN (~72 kDa), acting upon the 56 bp *telRL* target sequence that is entirely sufficient to confer TelN-mediated processing and linearization both *in vivo* and *in vitro*[19,20]. Similarly, phage PY54, isolated from *Yersinia enterocolitica*, maintains its prophage as a linear, circularly permuted, and covalently closed plasmid with telomere hairpin ends and a genome size of 46 kb. The paralogous minimal protelomerase target site of PY54 is a 42 bp perfect palindrome that unlike N15, only partially functions *in vivo* in the absence of adjacent sequences [21]. The paralogue of the N15 TelN protelomerase, Tel, encodes a 77 kDa protein with observably identical function, able to process recombinant plasmids containing the *pal*, 42 bp palindromic target site [21]. The *tel* gene possesses 60% sequence identity to *telN* and the active recombinases are similar in size (~77 kDa). In addition, there is a partial homology between the 42 bp PY54 *pal* site and the 56 bp N15 *telRL* site, where the ten central palindromic nucleotides (5'-TACGCGCGTA-3') are identical [21]. Despite obvious similarities between the two phages they are evolutionary quite distant, where N15 is more closely related to λ than to PY54. Purified TelN was shown to process circular and supercoiled plasmid DNA containing the identified target site, *telRL*, to produce linear double-stranded DNA with covalently closed ends. The lcc and mini lcc DNA vectors produced *in vitro* by recombinant TelN have been successfully applied in gene delivery experiments, and showed higher and more durable expression of the gene of interest in targeted human cells [20,21]. In contrast, to the best of our knowledge, there are no reported applications of the Tel-*pal* system. Furthermore, current TelN-*telRL* applications are based on recombinant TelN production for *in vitro* lcc DNA vector generation [22]. In this study, we report for the first time the development and characterization of an optimized *in vivo* mini lcc DNA production platform, exploiting the Tel-*pal* and TelN*-telRL* recombination systems. This one-step production system combines the biocompatibility benefits of “minicircles” with the transfection efficiency and safety profile of “MIDGE”.

## Results

### R-cells exhibit temperature-regulated recombinase expression

**Figure 1:**
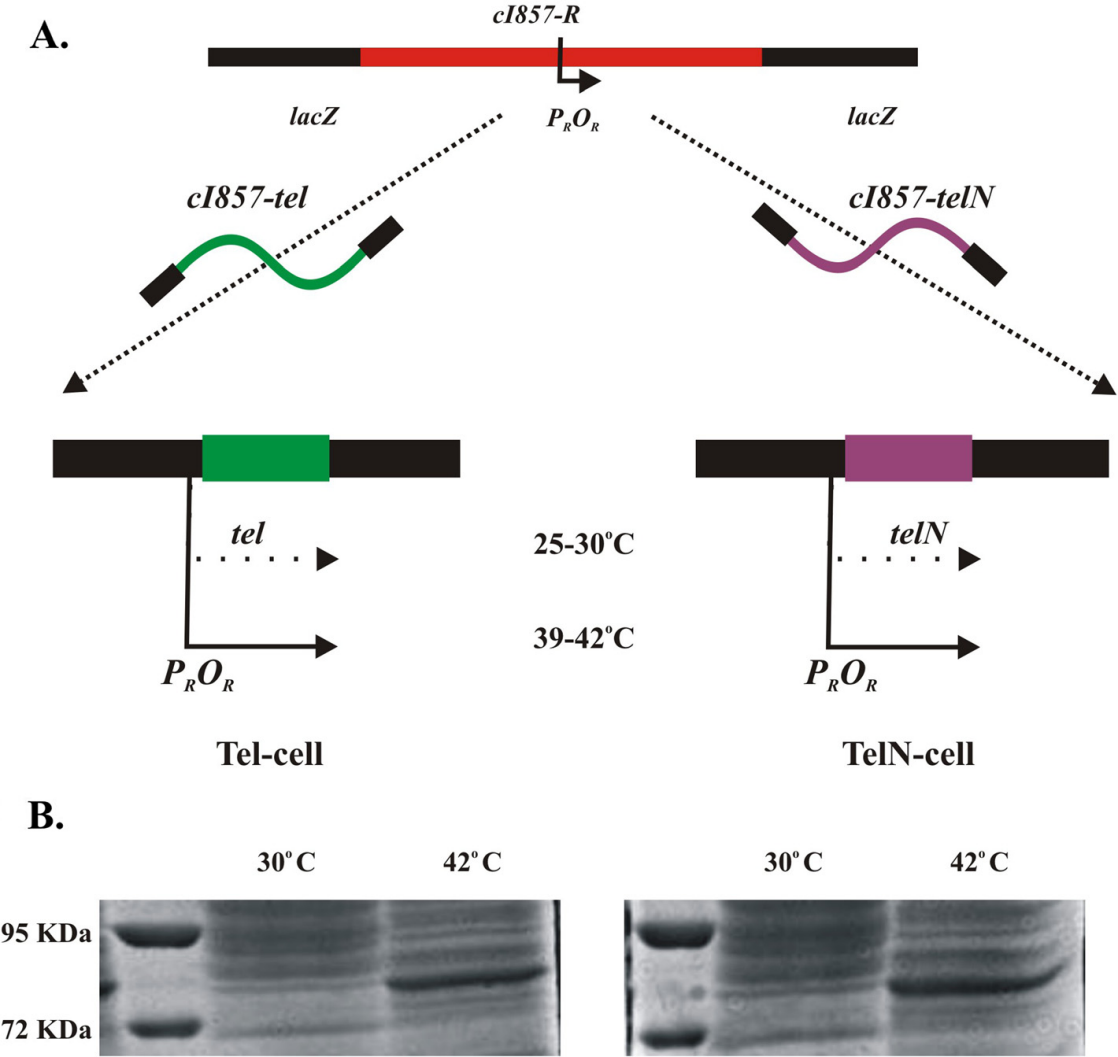
**R-cell construction and temperature-regulated expression of Tel and TelN.A**. R-cell construction and temperature-regulated expression of recombinases, Tel and TelN. The *cI857-R* cassette was inserted into the *lacZ* gene of *E. coli* by homologous recombination. Under repressed (25-30°C) conditions, the λ temperature-sensitive CI857 repressor binds to λ operators to inhibit transcription of the recombinase gene, but upon shifting to 40-42°C, the repressor is denatured, falling off the operators and induces expression of the recombinase from the strong λ *pL/pR* promoter. **B**. Controllable Tel and TelN expression from R-cells. Expression of the recombinase proteins Tel and TelN under repressed (30°C) and induced (42°C) conditions from total R-cell extract. Lane 1: Weight marker; Lane 2: Recombinase.

Recombinant *E. coli* cells (R-cells) were constructed that place *tel* or *telN* recombinase genes under control of the bacteriophage λ strong promoter, *pL,* that is regulated by the temperature-sensitive λ repressor, CI[Ts]857 (Figure 1A). We also similarly constructed *cre-*expressing cells to serve as a positive control, where *in vivo* Cre-*loxP* activity is well documented [23,24]. We examined total cellular protein in *tel* and *telN* R-cells under repressed (30°C) and fully induced (42°C) conditions (Figure 1B). As expected, both R-cells demonstrated minimal recombinase protein levels, identified at 72 kDa for both TelN and Tel at 30°C, where CI[Ts] actively binds the *oL* operator and represses transcription of the downstream recombinase gene. Upon shifting cells to 42°C, where repressor activity is completely abrogated and occlusion of *pR* and *pL* promoter activity is relieved, prominent recombinase expression was observed. These results confirm that the constructed Tel^+^ and Tel-N^+^ cells are temperature inducible for recombinase production.

**Figure 2:**
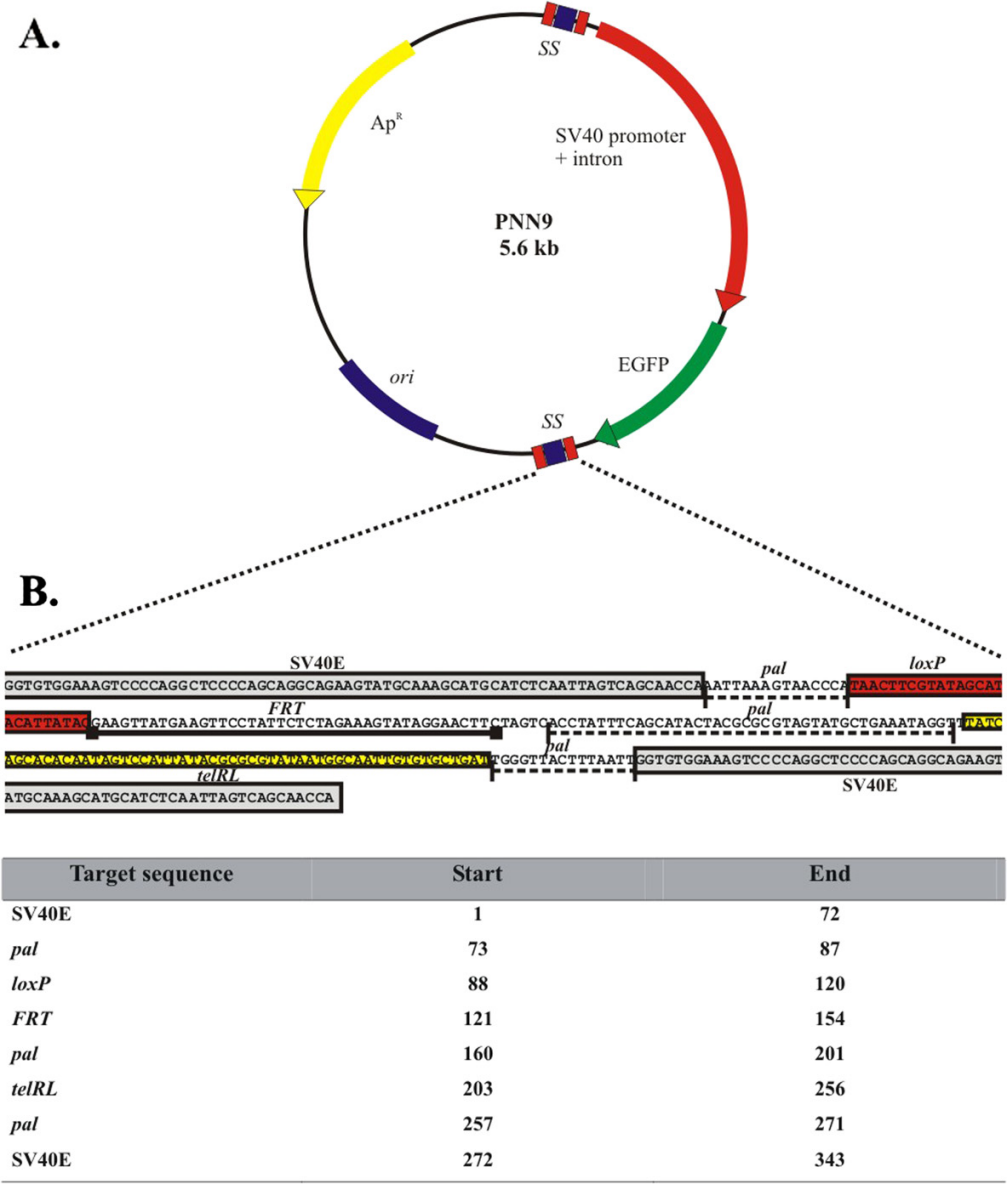
**Parent plasmid pNN9 and "Super Sequence" multi-target site.A**. Map of the parent pNN9 construct. The map denotes location of primary genetic elements, including the designed and integrated Super Sequence (SS). **B**. Map of the Super Sequence construct. The map includes relative locations of SV40 enhancer sequences as well as the *telRL, loxP* and *FRT* sequences integrated into non-binding regions of the 142 bp *pal* target site (Tel).

**Figure 3:**
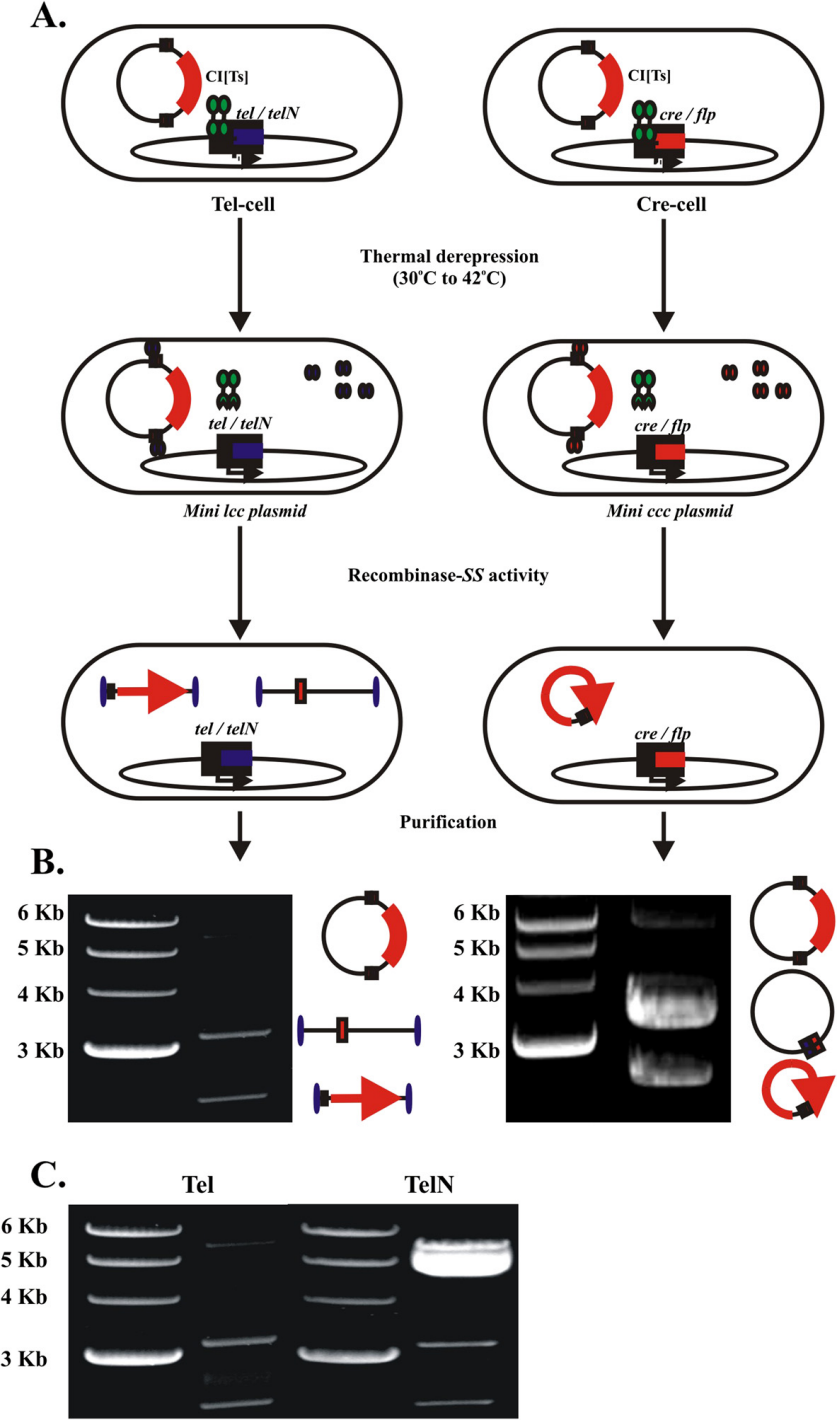
**Conditional processing of the parent plasmid DNA vectors.A**. R-cell conditional processing of the parent pNN9 plasmid into mini vectors. Under induced conditions, R-cells lead to the production of mini lcc (TelN- or Tel-cell) and mini ccc (Cre-cell) DNA vectors by recombinase activity on its target site encoded within the 2 Super Sequence sites on pNN9. Processing of the parent plasmid DNA results in production of two species—the mini DNA vector and the mini plasmid backbone. **B**. Processing of the parent plasmid construct into mini lcc and mini ccc vectors. Efficiency of processing of the pNN9 plasmid into mini lcc DNAs (Tel) and “minicircles” (Cre) after plasmid extraction from R-cells under induced (42°C) conditions. Schematics adjacent to each bands show the DNA constructs and expected conformation. **C**. *In vivo* Tel-*pal* recombination efficiency versus TelN-*telRL*. Efficiency of processing of the pNN9 plasmid into mini lcc DNA vectors in Tel^+^ versus TelN^+^ R-cells.

In combination with the R-cell system, we next constructed a eukaryotic *egfp*-expression plasmid DNA vector that carries two specialized 343 bp sequences placed upstream of the SV40 promoter and downstream of the polyadenylation signal of the minimal *egfp* expression cassette (pNN9; Figure 2A). This construct, termed “Super Sequence” (SS) carries a modified *pal* target sequence of Tel, with integrated *telRL* (TelN), *loxP* (Cre) and *FRT* (Flp) sequences in non-binding regions of *pal* sequence, and SV40 enhancer sequences that flank *pal* on either side to enhance nuclear translocation (Figure 2B). The rationale behind the multi-target sequence composition of SS was to allow the same parent plasmid vector to be passed through different R-cells to generate either mini lcc or mini ccc conformation of the minimal expression cassette. We employed the pNN9 (5.6 kb) plasmid to confirm that controlled production of recombinase from R-cells was associated with controllable recombinase enzymatic activity, by examining the ability of R-cells to convert plasmids carrying the SS sites to the appropriate mini lcc (Tel or TelN) or mini ccc (Cre) conformation. The SS^+^-derivative (pNN9; Table 1) was passaged through Tel/TelN^+^ or Cre^+^ R-cells at optimized conditions and assessed formation of mini lcc or mini ccc DNA vectors during the one-step *in vivo* processing and amplification protocol (Figure 3A). By flanking the gene of interest (GOI) expression cassette with two SS sites, we expected to excise the GOI (2.4 kb) for easy separation from the remaining prokaryotic backbone (3.2 kb) parental DNA, including the origin of replication and Ap^R^ marker at temperatures inducing recombinase expression (Figure 3B). Recombinase activity on pNN9 was evident when the plasmid was processed at 42°C where R-cell recombinase expression was “on”. No SS^+^ plasmid processing was evident at 30°C when expression was “off”, or with the SS^—^ control plasmids under all tested conditions (data is not shown). Optimal linearization conditions for pNN9 were conducted in both Tel^+^ and TelN^+^ cells and pNN9 processed products were extracted and analyzed. During the optimization process, different thermal conditions were tested to determine the ideal induction stage during R-cells growth and the induction duration that generates maximum recombinase production and plasmid processing. We found that shifting the temperature at late logarithmic phase to 42°C for 30 min to induce R-cells conferred optimal recombinase expression and plasmid processing. In all cases, extracted mini lcc DNA vectors behaved with equivalent stability in storage and manipulation to their ccc counterparts or parental plasmid DNA (data not shown). Linearization of pNN9 and extraction of the mini lcc vector, was also much more efficient in Tel^+^, as compared to the TelN^+^ counterpart (Figure 3C). These findings indicate that Tel is active upon *pal*^+^ constructs *in vivo* and that the modification of *pal* target site through the addition of *loxP, FRT,* and *telRL* minimal target sequences into non-binding regions of the full 142 bp *pal* target site does not abrogate Tel*-pal* functionality.

**Table 1:** Bacteria, Phage and Plasmids

**Strain**	**Genotype or description**	**Source**
***Bacteria***		
BW23474	F-, *Δ(argF-lac)169*, *ΔuidA4::pir-116*, *recA1*, *rpoS396*(Am), *endA9*(del-ins)*::FRT*, *rph-1*, *hsdR514*, *rob-1*, *creC510*	*E. coli* Genetic Stock Center (CGSC) # 7838 [39]
DH5α	F-, *Δ(argF-lac)169*, φ80d*lacZ58*(M15), *ΔphoA8*, *glnV44*(AS), *λ*^*-*^, *deoR481*, *rfbC1*, *gyrA96*(NalR), *recA1*, *endA1*, *thi-1*, *hsdR17*	CGSC # 12384
DH5α λpir	F-, *Δ(argF-lac)169*,*endA1*, *pir*^*+*^, *recA1*	Gift from Dr. T. Charles; [40]
JM109	F', *Δ(gpt-lac)0*, *glnV44*(AS), *λ*^*-*^, *rfbC1*, *gyrA96*(NalR), *recA1*, *endA1*, *spoT1*?, *thi-1*, *hsdR17*, pWM5, F128-x	New England Biolabs
W3101	F-, *λ*^*-*^, *galT22*, *IN(rrnD-rrnE)1*, *rph-1*	CGSC # 4467;
W3110	F-, *λ*^*-*^, *IN(rrnD-rrnE)1*, *rph-1*	CGSC # 4474;[41]
W3110-Cre (W1NN)	F-, *λ*^*-*^, *IN(rrnD-rrnE)1*, *rph-1 lacZ::cat-cI857-cre* (Cm^R^)	This study
W3110-TelN (W2NN)	F-, *λ*^*-*^, *IN(rrnD-rrnE)1*, *rph-1 lacZ::cat-cI857-telN* (Cm^R^)	This study
W3110-Tel (W3NN)	F-, *λ*^*-*^, *IN(rrnD-rrnE)1*, *rph-1 lacZ::cat-cI857-tel* (Cm^R^)	This study
***Phages***		
N15	Wild type (wt) (*telN*^*+*^, *tos*^*+*^)	Gift from Dr. S. Hertwig; [21]
P1	wt (*cre*^*+*^, *loxP*^*+*^)	Gift from Dr. B. Funnell; [42]
PY54	wt (*tel*^*+*^, *pal*^*+*^)	Gift from Dr. S. Hertwig; [21]
***Plasmids***		
pAH120	*attP* λ integration plasmid (Kn^R^)	NBRP [25]
pAH123	*cI857-pL-int* Φ80 (Ap^R^)	NBRP [25]
pAH153	*attP* Φ80 integration plasmid (Kn^R^)	NBRP [25]
pBRINT	*lacZ::cat-MCS::lacZ* (Cm^R^)	NBRP; [37]
pGL2	SV40P-Luc-PolyA-SV40 intron	Promega
pInt(ts)	*cI857-pL-int* λ (Ap^R^)	NBRP [25]
pNN1	*cI857-pR-pL-cre-tL* (Ap^R^)	This study
pNN2	*cI857-pR-pL-tel-tL* (Ap^R^)	This study
pNN3	*cI857-pR-pL-telN-tL* (Ap^R^)	This study
pNN4	*lacZ::cat- cI857-pR-pL-cre-tL::lacZ* (Cm^R^)	This study
pNN5	*lacZ::cat- cI857-pR-pL-tel-tL::lacZ* (Cm^R^)	This study
pNN6	*lacZ::cat- cI857-pR-pL-telN-tL::lacZ* (Cm^R^)	This study
pNN7	*pGL2-egfp switched for luc*	This study
pNN8	pNN7 + SS (upstream of SV40 promoter)	This study
pNN9	pNN8-SS (2SS) (second SS downstream of SV40 polyA sequence)	This study
pNN10	pAH120 (SS^+^)	This study
pNN11	pAH153 (SS^+^)	This study
pPL451	*cI857-pR-pL-MCS-tL* (Ap^R^)	Accession # AB248919 National Bioresource Project (NBRP); [43]

### Integration of lcc DNA vector into the chromosome results in loss of cell viability

**Figure 4:**
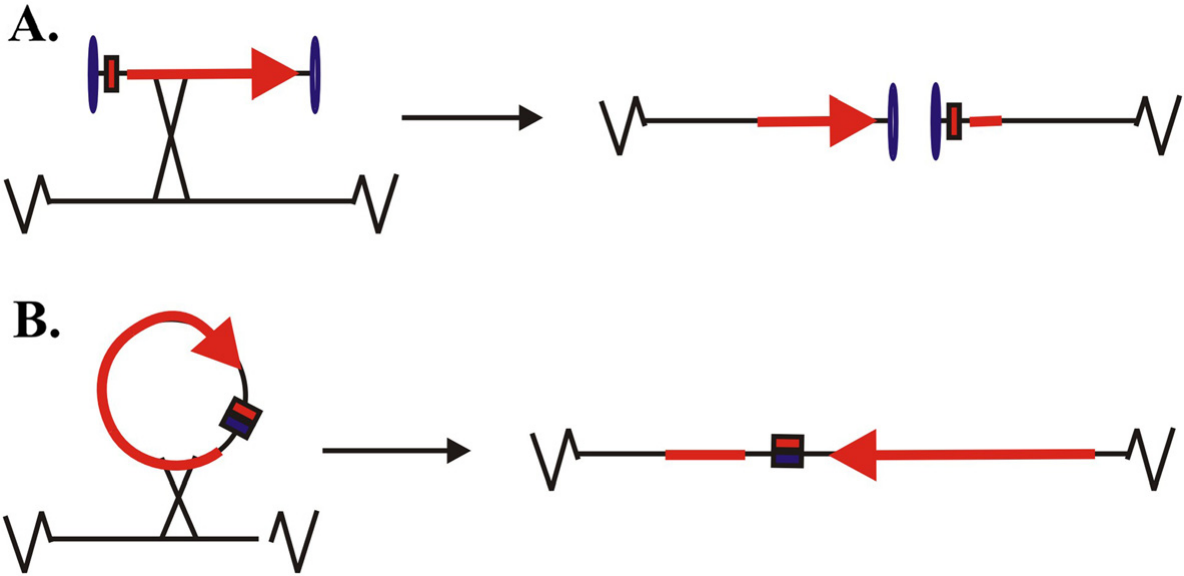
**Schematic representations of linear and circular vector integration events.** A mini vector that undergoes a single recombination event with the host chromosome is rare due to the removal of all elements except the cistron containing the GOI expression cassette and the flanking Super Sequence sites. **A**. Lcc plasmid DNA vector integration event would result in chromosomal ”break” at the site of integration, whereby the chromosome cannot be replicated or segregated and the integrated mammalian cell cannot divide due to separation of centromere from telomere. **B**. A “minicircle” vector can integrate into a non-essential region of the host chromosome without “breaking” the chromosome, whereby the cell is able to continue dividing with the insertion.

We asked whether a single crossover recombination event integrating a linear pDNA vector with covalently closed ends into a host cell chromosome would disrupt the chromosome and kill the cell (Figure 4). To assess the outcome of linearizing the *E. coli* chromosome by lcc DNA integration, we employed an *in vivo* approach by exploiting λ and Φ80 Int-*att* site-specific recombination systems [25] in a Rec^+^ background. To target the chromosome, an integrating plasmid vector that carries the λ *attP* target site of the λ integrase and an R6K origin was used as it is only capable of replication in the presence of π protein (encoded by *pir* gene), which is absent from all R-cells. As such, the plasmid is either integrated or rapidly lost from the growing R-cell population. An SS was incorporated into the integrating pDNA vector to change plasmid conformation from ccc to lcc in the presence of TelN or Tel in R-cells. Using this system we sought to assess integration efficiency following transformation of parent, Tel^+^ or TelN^+^ R-cells by SS^—^ and SS^+^ integrating plasmid vectors under conditions induced or repressed for recombinase expression in R-cells. A ccc λ or Φ80 *attP*^*+*^ SS^+^ plasmid that is taken up by the cell expressing λ or Φ80 *int*, respectively, and producing Tel or TelN, should be altered from ccc to lcc conformation. Upon Int-mediated *att*-specific chromosomal integration, the recombination of lcc should disrupt the chromosome. And, as unintegrated plasmids are incapable of replication, they would be rapidly lost from the cell population.

The integration frequency (IF) of λ *attP* SS^+^ integrating plasmids into induced Tel^+^R-cells was more than 10^5^-fold lower than that seen for the SS^—^ counterpart or the SS^+^ integrating plasmids in parent W3110 cells (Table 2). TelN^+^ R-cells did not demonstrate nearly as strong an effect, whereby IF compared to the wild type cells was 50-fold lower, and only 25-fold lower versus integration of the SS^—^ derivative. We further studied up to 20 survivor isolates from both TelN^+^ and Tel^+^ cells by PCR for evidence of chromosomal linearization, and found that 100% of tested survivors possessed intact (unlinearized) plasmid. These findings are likely due to the a higher efficiency of Tel*-pal* system in formation of a lcc product as compared to TelN acting on the minimal *telRL* target site encoded within the SS. Although we attempted to repeat this experiment using a constructed Φ80 *attP* SS^+^ integrating pDNA vector by the R-cells expressing Φ80 *int*, we found that integration frequencies were lower across all tested cells.

**Table 2:** Linear covalently closed (lcc) plasmid confers reduced integration frequency

**R-cell**^**1**^	**Plasmid SS (+/-)**^**2**^	**Integration Frequency**^**3**^
**Cre**	—	0.76
**Cre**	+	0.005
**TelN**	—	0.51
**TelN**	+	0.02
**Tel**	—	1.0
**Tel**	+	1.03 X 10^-5^

^1^ R-cells are all W3110 derivatives expressing recombinase gene at 42°C the recombinase gene is fully induced . R-cells carry the λ *int* expression plasmid pAH153.

^2^ pAH120 plasmid carrying λ *attP*.

^3^ Mean of minimum 3 trials. IF is expressed as fraction of integration frequency of parent strain, W3110.

We next sought to investigate chromosomal linearization by constructing SS^+^ or SS^—^ integrants using the same λ and Φ80 Int*-attP* plasmid integration system. Unlike the previous experiment, this time, rather than assessing the number of integrants formed in the presence of the linearizing recombinase, we first stably integrated the plasmid into the chromosome before inducing recombinase expression. Cells carrying the integrated SS^+^ or SS^—^ vector were maintained under repressed *tel* and *telN* conditions (30°C) and several isolates were assessed for integration of single versus multiple copies of plasmid by PCR. In all cases, single integration events represented the majority of recombinants for both SS^+^ and SS^—^ λ or Φ80 *attP* integrating plasmids (56.5-100%). Multiple copy integrants were not studied further and discarded.

To determine the fate of cells upon linearizing/disrupting the *E. coli* chromosome, we incubated λ site-specific SS^—^ and SS^+^ integrants under conditions that provide no to very low, or fully derepressed expression of the recombinase at 30°C and 42°C, respectively, and then measured cell viability (Table 3). Under repressed conditions (30°C), all recombinant cells retained near full viability regardless of the presence or absence of the SS integrated in the chromosome. However, upon shifting temperature to 42°C and inducing expression of Tel or TelN, recombinants that carried SS^+^ showed dramatically reduced viability. And, in both systems, Tel-cells resulted in approximately 5-fold greater killing than that seen in TelN-cells. Interestingly, the killing effect of SS^+^ plasmid in Φ80 *attB* site was about 10-fold lower than was observed when integrated into λ *attB* site. This finding suggests that positioning of the *attB* site may influence the viability of cells with a linear chromosome.

**Table 3:** Recombinase-mediated linearization of the chromosome results in cell killing

**R-cell^1^**	**Integrated plasmid SS (+/−)**^**2**^		**Cell viability following induction **^**3**^	
			**30^o^C**	**42^o^C**
**TelN**	—	λ	0.8	0.6
**TelN**	+	λ	1.0	5.7 X 10^-4^
**TelN**	—	Φ80	0.8	0.4
**TelN**	+	Φ80	1.0	5 X 10^-3^
**Tel**	—	λ	1.0	1.0
**Tel**	+	λ	1.0	1.3 X 10^-4^
**Tel**	—	Φ80	1.0	0.8
**Tel**	**+**	Φ80	1.0	1.1 X 10^-3^

^1^ R-cells are all W3110 derivatives cured of pAH153 plasmid during construction stage and confirmed for single integration event by PCR.

^2^ pAH120 plasmid carrying λ *attP* grown and prepared under minimal inducing conditions (30°C).

^3^ Average of minimum 3 trials. Viability is expressed as a fraction of colonies counted under non-induced (25°C) conditions.

### Visualization of Cells upon induction

**Figure 5:**
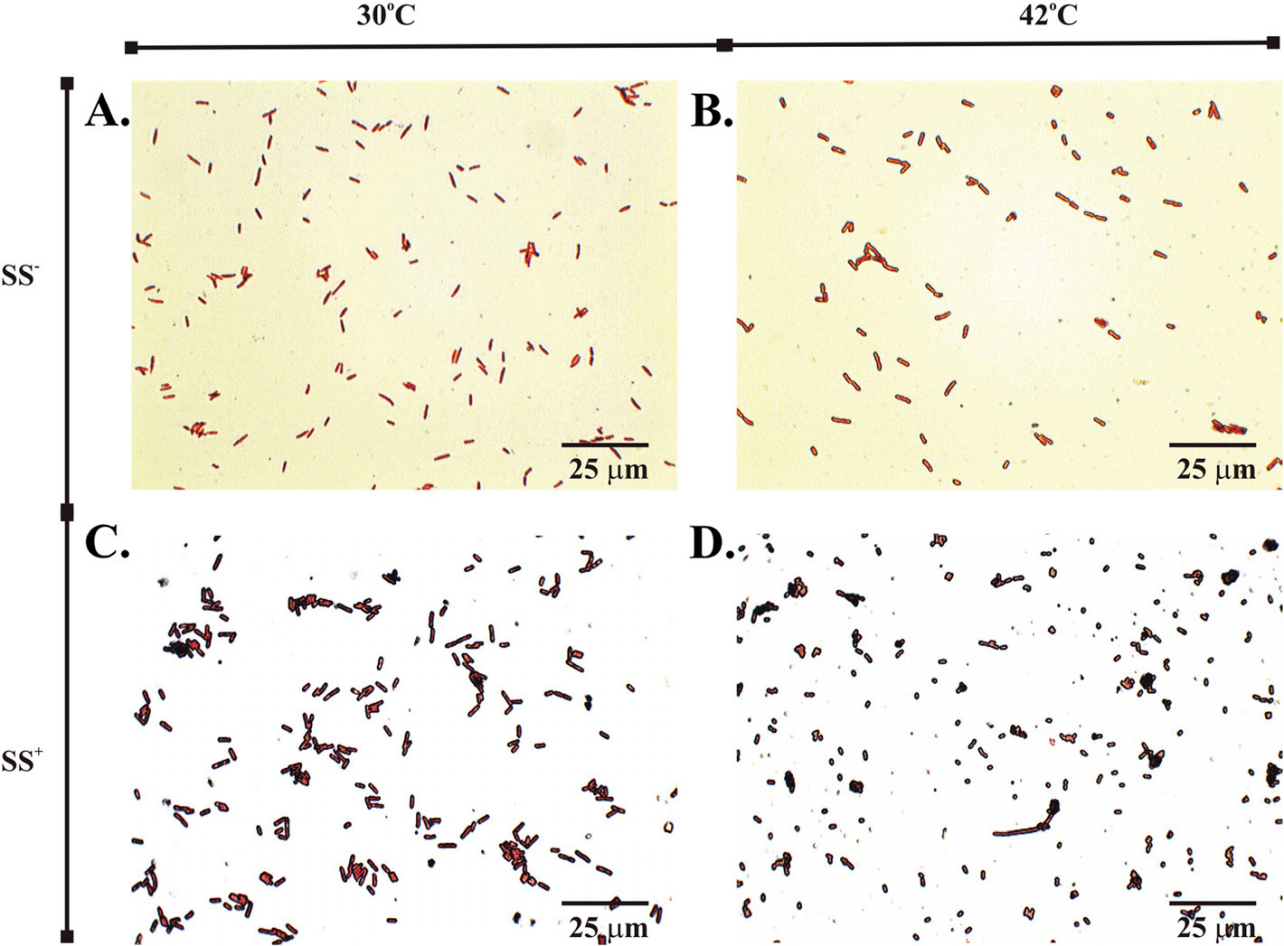
**Effect of Tel-mediated chromosomal disruption on cell morphology.** Tel-mediated disruption of the host chromosome results in a contracted cellular morphology. Tel^+^ R-Cells possessing an integrated plasmid that was SS^+^ (*pal^+^*) or SS^—^ (*pal^—^*) were grown in LB media at 30°C (no Tel production) to early log phase A_600_ = 0.2 , then were divided in two tubes and grown separately at 30°C or 42°C to reach to late log phase A_600_ = 0.8. Bacterial smears were then prepared on the slide and heat fixed and gram stained. Pictures of bacteria were taken at 1000X magnification. **A**. SS^—^ integrants at 30°C where *tel* expression is repressed; **B**. SS^—^ integrants at 42°C where *tel* is induced but the *pal* target site is absent; **C**. SS^+^ integrants at 30°C where *pal* target site is present but *tel* expression is repressed; **D**. SS^+^ integrants at 30°C where *tel* is induced and present to act on the chromosomally integrated *pal* target site.

Wild type and Tel-cells carrying chromosomally inserted SS^+^ or SS^—^ integrating plasmid vectors were gram stained and visualized under repressed (30°C) versus induced (42°C) conditions for *tel* recombinase expression to investigate cell morphology as a result of chromosomal disruption (Figure 5). At 42°C, only SS^+^ Tel^+^ integrants demonstrated a highly contracted and irregular morphology compared to SS^—^ cells, or SS^+^ cells grown at 30°C, repressed for *tel* expression. We screened 10 Tel^+^/SS^+^ colonies that grew at 42°C, for linearization by PCR and found that all of the colonies showed an unlinearized SS^+^ plasmid insert (data not shown). Surviving colonies were however, very small compared to their SS^—^ counterpart or the wild type parent, and retained this morphology whether grown under inducing or repressed conditions. We averaged the length of 400 randomly selected cells. We noted that the SS^—^ controls were similar at both 30^o^ and 42°C with a normal average length for log phase *E. coli* in rich medium, measuring 3.9 ± 0.4 and 3.9 ± 0.6 μm respectively. In contrast, cells, possessing the chromosomally integrated SS site, were much smaller at 42°C, where *tel* expression was fully induced, and averaging only 1.2 ± 0.2 μm in length. In contrast, a small proportion of these cells (1.5%) were filamentous. This may be attributed to replication delay or inhibition in these cells. Under the conditions where we would expect chromosomal disruption, we also observed a great deal of cellular debris. At 30°C, where *tel* expression was repressed, cells were considerably larger at 3.0 ± 0.8 μm but there was a much greater variability in size between cells ranging from 1.2 to 4 μm. This variability could be the consequence of leaky expression of *tel* due to incomplete repression of the CI[Ts]857 repressor at 30°C.

## Discussion

To date, mini DNA vector production has been limited to *in vitro* strategies, adding expense and complexity to the developmental process, particularly in scalability. The mini DNA vector production system, described here, is an *in vivo* platform to generate high quality bacterial sequence-free mini DNA vectors in both lcc and ccc topology. Modified mini vectors can be purified directly from our engineered *E. coli* cells (R-cells) using standard plasmid isolation methods, and without the need for digestion, ligation, and gel purification. To generate R-cells, we flanked the *cI*[Ts]857-protelomerase expression cassettes by homology to *lacZ* gene and recombined the cassette into *recA*^*+*^ W3110 *E. coli* cells screening for Lac^-^ colonies. R-cells were designed and optimized to chromosomally encode specific recombinases under control of a strong λ *pL* promoter. However, since strong promoters impart metabolic burden on the host cell, where protein production may occur at the expense of cell growth [26], we employed a simple thermally-regulated promoter system that circumvents any potential toxicity, metabolic stress or recombinase interference that might arise from the use of chemical-mediated induction strategies. As such we were able to optimize the production of mini DNA vectors through simple control over manufacture temperature using the λ CI[Ts]857 *oLpL* expression system [27]. Various modifications to our parent vector construct by simple passaging through different R-cells was made possible by the insertion of the unique multi-target sequence (Super Sequence) described for the first time here. The Super Sequence inserts the cleave-joining multi-target site for TelN, Cre, and Flp enzymes within the non-coding regions of the 142 bp minimal sequence of *pal* site required for *in vivo* processing by Tel [21]. The retained activity of Tel in processing this site indicates that the replacement of non-coding regions within the *pal* sequence of PY54 does not compromise cleave-joining activity of the Tel-*pal* system.

Mini linear covalently closed (lcc) DNA vectors, devoid of parental prokaryotic genetic elements were successfully generated *in vivo* exploiting the bacteriophage PY54-derived protelomerase recombination system. The mini vector produced via this system is a stable linear DNA expression cassette with covalently closed ends. Application of pDNA vectors in naked, lipoplexed, or polyplexed form for gene transfer conventionally employs plasmid vectors designed either to administer genes coding for therapeutic proteins, antigens, or antibodies into a given organism; or introduce a correct gene into a host cell to replace the mal- or non-functional allele [28]. Derived from conventional plasmid DNA vectors, bacterial sequence-free mini DNA derivatives provide superior alternatives to traditional plasmid vectors, with some advancing in clinical trials [29]. Conventional plasmid vectors carry a bacterial backbone in addition to the cistron expressing the gene of interest (GOI) in addition to necessary regulatory elements such as the promoter, enhancer, intron, and polyA terminal sequence. While necessary for amplification and maintenance in the prokaryotic host, the plasmid vector backbone carries prokaryotic genetic elements, including antibiotic resistance gene(s) and an origin of replication. It was previously shown that antibiotic resistance genes are undesirable for administration to human body due to potential adverse effects and events such as their horizontal gene transfer imparting resistance to naturally occurring mammalian host flora; and their compromising effect on GOI expression [30]. CpG motifs, 20 times more common in prokaryotic DNA, induce polygonal B-cell activation and activate Toll-like receptors that in addition to generating potentially unwanted immunostimulatory responses, may also reduce or abrogate transgene expression [30,31].

Bacterial sequence-free mini DNA vectors provide better bioavailability compared to conventional plasmid DNA vectors due to their smaller size and higher immuno-compatibility due to reduced or eliminated unwanted immune responses. Their smaller size confers higher transfection efficiency and higher copy numbers of the vector per unit mass, resulting in lower toxicity due to the need for less transfection reagents. Smaller pDNA vectors also better resist shear forces associated with the *in vivo* administration and delivery to the target site [32], important improvements to safety and efficiency of transgene delivery in treatment of human disease [33].

Conventional plasmid vectors are extra-chromosomal, circular, covalently closed, double-stranded DNA molecules that often possess elements or sequences such as viral promoters or cloned coding sequences that could subject them to unwanted recombination events [34]. A major safety concern associated with gene delivery, whether by viral or non-viral carriers, is the potential for integration of plasmid DNA into the host chromosome. Integration events that activate proto-oncogenes and/or deactivate tumor suppressor genes may result in oncogenesis or silencing of adjacent genes [35]. We showed here that the frequency of viable integrants of lcc pDNA vector into the circular prokaryotic *E. coli* genome is reduced at least five orders of magnitude compared to its ccc counterpart. The lethal effect of chromosomal disruption in *E. coli* was investigated by site-specific insertion of the lcc integrating pDNA vector using Int-*attB* systems from bacteriophages λ and Φ80. Our results linking site of chromosomal disruption to degree of lethality agree with previous work by Cui *et al*. ([2007]) who reported that the closer the linearization of the chromosome occurred to the *E. coli* origin of replication, the stronger was the observed growth defect [36]. Our microscopy results indicated a dramatic reduction in cell size and surrounding cellular debris following genome disruption, which we attribute to replication inhibition and associated cellular stress, phenotypes we are presently investigating further in bacterial and mammalian cells. The generation of filamentous cells similarly may be due to SOS-independent filamentation arising from inhibited or dramatically slowed replication [36].

Considering the lethal effect of lcc DNA vector integration into host chromosome, lcc based gene replacement vectors will likely provide a safer option for targeted recombination events. We report elsewhere that lcc vector integration into human cells results in chromosomal disruptions and cell death, preventing propagation of the lcc vector-integrated cell and its natural elimination from the transfected cell population. As such, mini lcc vectors potentially serve as ideal choice for knock-in and gene replacement studies in stem and other pluripotent cells for the generation of transgenic plant and animal models and regenerative medicine, avoiding damage and side effects of integration.

## Conclusion

Mini lcc DNA vector constructs were generated *in vivo* exploiting the bacteriophage PY54-derived Tel-*pal* recombination system in a conditional recombinase expression scheme. The mini lcc DNA vectors provide a safer alternative to conventional pDNA vectors without compromising utility. In addition to a unique multi-target sequence, each vector is equipped with two SV40 enhancer sequences at the two covalently closed ends of the linear plasmids to facilitate the nuclear uptake and enhance the transfection efficiency and expression of the GOI. The production system reported here and its associated safety profile may serve as a basis for simplified, scalable, and safer DNA vector production that could drive the design of virtually limitless innovations with applications to health, agriculture, and industry.

## Methods

### Strains and plasmids

*E. coli* K-12 strains were used in the generation of all recombinant cell constructs and DH5α and JM109, in particular, were employed as hosts for plasmid constructions and amplification.

A list of bacterial and phage strains used in this study are shown in Table 1.

### Construction of Recombinant Cells (R-cells)

W3110 was used for chromosomal engineering studies and *in vivo* recombinase expression as follows. Protelomerase coding gene *tel* was amplified from bacteriophage PY54 lysate using the following primers: Tel-F 5^′^-GC*GGATCC*TGGGTTACTTTAATTTGTGTGTT-3^′^ and Tel-R 5^′^-CG*CTCGAG*TTACTCCATATTTTCAGTCCATGCTTGT-3^′^ (annealing Tm 64°C). Protelomerase coding gene *telN* was amplified from bacteriophage N15 lysate using primers: TelN-F 5^′^-ATC*GGATCC*CGATATCCAGAGACTTAGAAACGGG-3^′^ and TelN-R 5^′^- ATATA*AAGCTT*CTTTTAGCTGTAGTACGTTTCCCATGCG-3^′^ (annealing Tm 62°C). As a positive control for *in vivo* production of modified DNA vectors, the recombinase encoding gene *cre* was amplified from bacteriophage P1*rev6* lysate using primers: Cre-F 5^′^-G*GAATTC*CGGTCGCTGGCGTTTCTATGAC-3^′^ and Cre-R 5^′^-CG*CTCGAG*TGAATATTAGTGCTTACAGACAG-3^′^ (annealing Tm 66°C). Italicized regions denote restriction sites for *BamH*I, *Xho*I, *Hind*III, and *EcoR*I. PCR amplifications were conducted using Phusion Flash High-Fidelity PCR Master Mix (New England Biolabs) for 30 s at 98°C for initial denaturation, 30 cycles of 5 s at 98°C, 10 s at annealing Tm, 45 s at 72°C, and 2 min at 72°C for final extension to generate *cre* (1.3 kb), *tel* (2.1 kb), and *telN* (2.3 kb) fragments. Constructs were tested and confirmed by colony PCR and analytical digestion. PCR products were purified from 0.8% agarose gel (Qiagen Gel extraction kit), and digested with the listed enzymes (New England Biolabs). Recombinase genes were cloned into the MCS of the inducible prokaryotic expression plasmid vector pPL451 (Accession #AB248919) to produce pNN1, pNN2, and pNN3 vectors. pPL451 (4.2 kb) imparts temperature-regulated expression of the cloned gene via CI[Ts]857-mediated repression of the λ *pL* strong promoter. A list of plasmids used or constructed in this study is shown in Table 1. All primers were designed using the Gene Runner 3.01 (Hastings Software, Inc) and synthesized commercially (Sigma-Aldrich, Inc). R-cells were constructed via insertion of recombinase genes into *E. coli* W3110 chromosome using the pBRINT-*cat* integrating plasmids, which facilitate the homologous recombination and chromosomal integration of cloned sequence of interest into the *lacZ* gene of *E. coli*[37]. For each plasmid construct encoding inducible expression of a cloned recombinase in pPL451, the *cI857-P*_*L*_*-X-t*_*L*_ cassette (where X = *cre, tel* or *telN*) was amplified from the pNN1 to 3 constructs by the cI857X-F 5^′^-TCC*CCGCGG*AGCTATGACCATGATTACGAATTGC-3^′^, cI857telN/cre-R 5^′^-GG*ACTAGT*CCCCATTCAGGCTGCGCAACTGTTG-3^′^, and cI857tel-R 5^′^-GC*TCTAGA*GCAGGCTGCGCAACTGTTGGGAAG**-**3^′^ primers with *Sac*II, *Spe*I, and *Xba*I sites respectively. The amplified cassettes were cloned into the MCS of pBRINT (Cm^R^) integrating plasmid to produce pNN4, pNN5, and pNN6 integrating pDNA constructs. Amplification have been performed by the Phusion Flash High-Fidelity PCR Master Mix (New England Biolabs) for 10 s at 98°C for initial denaturation, 30 cycles of 1 s at 98°C, 5 s at 68°C, 120 s at 72°C, and 1 min at 72°C for final extension to generate cI857*-cre* (2.8 kb), cI857-*tel* (3.2 kb), and cI857*-telN* (3.5 kb) fragments. Constructs were tested and confirmed by colony PCR and analytical digestion.

*E. coli* cells were grown in Luria–Bertani (LB) medium and plated on LB-Agar plates composed of 1.0% Tryptone, 0.5% Yeast Extract, 1.0% NaCl, pH 7.0. Antibiotics (Ab) (Sigma-Aldrich, Inc) were used at the following concentrations for the growth of cells carrying multi-copy plasmids: ampicillin (Ap, 100 μg/ml in H_2_O), chloramphenicol (Cm, 25 μg/ml in isopropanol), gentamycin (Gm, 15 μg/ml in H_2_O), and kanamycin (Km, 50 μg/ml in H_2_O). H_2_O used for dilution of primers, plasmids, antibiotics, and production of competent cells is nuclease-free sterile molecular grade water (Sigma-Aldrich, Inc). To achieve chromosomal integration of the pNN4 to 6 constructs into Rec^+^ W3110, a 1:100 dilution of fresh overnight cells was grown on SOB media (2.0% Tryptone, 0.5% Yeast Extract, 2.0% NaCl, 1.0% KCl, 1.0% MgCl_2_ , and 1.0% MgSO_4_) at 37°C to A_600_ = 0.4 - 0.6 and cells were harvested by centrifugation at 5K RPM for 5 min. Cells were washed in water three times and W3110 cells were transformed by 1 μg of pNN4, 5, or 6 integrating vectors via electroporation at 800 v. Cells were recovered in SOC (SOB with 2.0% Glucose) at 30°C for 1 h, then spread onto selective media on 12.5 μg/ml of chloramphenicol, 100 μg/ml of 5-bromo-4-chloro-indolyl-β-D-galactopyranoside (X-Gal) (Promega), and 0.1 mM of Isopropyl β-D-1-thiogalactopyranoside (IPTG) (Sigma-Aldrich, Inc) added to LB agar plates and incubated overnight at 30°C. To make a 50 mg/ml stock solution, 500 mg X-Gal were dissolved in 10 ml dimethylformamide. Protect plates from light. White colonies were selected and further screened for sensitivity to ampicillin and chloramphenicol. White Ap^S^ colonies indicated loss the pNN integrating plasmids after disruptive insertion of the *cI857-P*_*L*_*-X-t*_*L*_ cassette into the *lacZ* gene, generating recombinant derivatives, W1NN, W2NN, and W3NN (Table 1). W3110 [*lacZ::cat- cI857(cre/tel/telN*)] recombinants were confirmed for presence of the *cI857-P*_*L*_*-X-t*_*L*_ cassette and temperature-regulated, conditional expression of recombinanse, by colony PCR using Taq polymerase enzyme (NEB), sequencing (Sigma), and SDS PAGE (BioRad) at various temperatures between 30°C and 42°C. For the selection of cells carrying the antibiotic selection markers integrated in the chromosome, the following concentrations were used: Cm (12.5 μg/ml), Gm (5 μg/ml), and Km (20 μg/ml).

### Construction of modified/ new generation of pDNA Vectors

The multi-purpose target site, named Super Sequence (SS), was designed to carry Cre, Flp and TelN minimal targets sites (*loxP-FRT-telRL*) respectively, all within the Tel 142 bp target site, *pal*. SS also carries a 78 bp SV40 enhancer sequence that flanks each side of the *pal* sequence to facilitate nuclear translocation and enhancing transfection efficiency. The SS fragment was synthesized by the GeneScript and cloned into the pUC57 by *Eco*RI and *Hin*dIII. Commercial eukaryotic expression plasmid vector, pGL2-promoter (5.8 kb) (Promega), was modified by replacement of the *luc* gene (1.65 kb) with *egfp* (790 bp) from pGFP (Clontech, Inc.) to form pNN7 (Genescript, Inc.) (4.9 kb) Next, SS was cloned immediately upstream of the SV40 promoter + intron site of pNN7 to form pNN8 (5.3 kb). Then the SS fragment was cloned downstream of the poly A site of pNN8 to form pNN9 (5.6 kb). The multi-copy pDNA vector pNN9 carries 2 SS sites that flank the *egfp* gene cassette and can be converted to a “minicircle” DNA vector (mediated by Cre-*loxP;* Flp*-FRT*), or a mini linear covalently closed DNA vector (mediated by TelN-*telRL;* Tel*-pal*). R-cells were transformed by 1 μg of pNN7 to 9 DNA constructs on LB + Ap (50 μg/ml) to A_600_ = 0.6 at 30°C with aeration. To induce recombinase expression and plasmid conformational conversion, transformed R-cells were heat shocked to induce the recombinase expression at 42°C for 30 min at mid-logarithmic phase of bacterial growth, before being transferred to 30°C overnight. Cells were then harvested and plasmid extracted (Omega kit, VWR). Plasmid topology was assayed by agarose gel electrophoresis and digestion. Standard recombinant DNA techniques were performed as described by Sambrook *et al.* (1989).

### Chromosomal Integration Assays of Linear Covalently Closed (LCC) DNA

“CRIM (conditional-replication, integration, and modular)” integrating plasmids [25] that possess a R6K origin of replication and a phage attachment (*attP*) site were modified to carry the SS fragment. SS was cloned into the pAH120 and pAH153 constructs (from NBRP; Table 1) by *Cla*I and *Bam*HI (New England Biolabs) to generate the pNN10 (3.3 kb) and pNN11 (2.6 kb) constructs, respectively. Plasmids were integrated into the host bacterial attachment (*attB*) site by supplying phage integrase (Int) from the helper plasmids. Plasmid pNN10 and pNN11 constructs were amplified in DH5α(λ*pir*) or BW23474, and the successful clones were confirmed by restriction pattern digestion and colony PCR. Int helper plasmids pINT-ts (λ *int*) and pAH123 (Φ80 *int*) that express *int* from λ *pL* under CI[Ts]857 control and carry a temperature sensitive pSC101 *ori* were used for integration of CRIM, pNN10 and pNN11 plasmids into their corresponding chromosomal *attB* sites of *pir*^*—*^ hosts that are non-permissive for plasmid replication.

R-Cells W1NN, W2NN, and W3NN (Table 1) W3110 *lacZ::cat- cI857(cre/tel/telN*)] were grown in 2 ml of SOB cultures at 30°C to an optical density of A_600_ = 0.6 and then electroporated and transformed by 50 ng helper plasmids pINT-Ts (λ *int*) or pAH123 (Φ80 *int*) and selected on LB + ampicillin agar at 30°C. R-Cells carrying helper plasmids were grown in 50 ml of SOB + ampicillin at 30°C to A_600_ = 0.6 then transformed by 1 μg of pAH120, pNN10 (pAH120+SS), pAH153, pNN11 (pAH153+SS) DNA, suspended in SOC at 37°C for 1 h for recovery and Int expression and at 42°C for 30 min for lose of helper plasmid, then selected on LB + antibiotic (15 μg/ml Km for pAH120, pNN10 and 5 μg/ml Gm for pAH153, pNN11) and incubated overnight at 37°C. Positive bacterial growth on selective media (15 μg/ml Km, or 5 μg/ml Gm) and negative bacterial growth on ampicillin media represents stable integration of the gene of interest and loss of the helper Int expression plasmid. Single-copy integrants W4NN to W15NN represent the SS^+^ pAH120 (pNN10), SS^-^ pAH120, SS^+^ pAH153 (pNN11), and SS^-^ pAH153 pDNA integrated into the W3110 or W3110 *lacZ::cat-cI857(cre/tel/telN*)]) were screened and selected by PCR using predesigned primers [25].

### Viability Assays of Linear Covalently Closed (LCC) DNA integration

Single copy pAH120, pNN10 (pAH120+SS), pAH153, pNN11 (pAH153+SS) DNA integrants W4NN to W15NN were isolated on selective media.

Integrants were grown in 2 ml LB media + selective antibiotic at 30°C to an optical density of A_600_ = 0.4 and then divided into two groups of 1 ml each and grown to A_600_ = 1. The first group was grown at 30°C with repressed *cre/tel/telN* expression and the second group was grown at 42°C, induced for *cre/tel/telN* expression. Cells were spread on selective plated and grew overnight at 30°C and 42°C, respectively. Viability was assayed by colony counting and size of the colonies grew at 30°C versus 42°C.

### Visualization of cells

Cells were visualized by gram staining as previously described [38]. Briefly, integrated cells were grown in 2 ml of LB media + selective antibiotic from freshly grown cells at 30°C to early log phase A_600_ = 0.2 and then were divided in two tubes and grown at 30°C and 42°C to late log phase, A_600_ = 0.8. Bacterial smears were then prepared on the slide and heat fixed and gram stained. Pictures of bacteria were taken at 1000X magnification. From pictures, 400 random cells were chosen for measurement under all tested conditions.

## Abbreviations

Bp: Base pair(s); CCC: Circular covalently closed; DTS: DNA nuclear targeting sequence; GOI: Gene of interest; IF: Integration frequency; kb: Kilo-base(s); kDa: KiloDalton(s); LB: Luria-Bertani medium; LCC: Linear covalently closed; MCS: Multiple cloning site(s); MIDGE: Minimalistic immunogenic defined gene expression; NLS: Nuclear localization signal; NPC: Nuclear membrane pore complex; *o*: Operator; PAGE: Polyacrylamide gel electrophoresis; pDNA: Plasmid DNA; *p*: Promoter; protelomerase: Prokaryotic telomerase; R: Recombinant cells; SDS: Sodium dodecyl sulfate; SS: Super Sequence; [ ]: Denotes plasmid-carrier state.

## Competing interests

The authors declare that there is no conflict of interests in the submission of this manuscript.

## Authors’ contributions

NN conducted the experimental work and significantly toward the design and writing of this manuscript. RAS designed the system described here and contributed significantly to the direction of the project and writing of the manuscript. Both authors read and approved the final manuscript.

## Authors’ information

RAS is currently an Assistant Professor of Pharmaceutical Science at University of Waterloo, School of Pharmacy and SDM Professor of Business and Entrepreneurship. NN is currently a PhD candidate at University of Waterloo.
